# The Applicability of Haarlem Integrated Diagnostic System in Diffuse Glial Tumors and Molecular Methods Affecting Prognosis

**DOI:** 10.4274/balkanmedj.galenos.2018.2018.1221

**Published:** 2019-07-11

**Authors:** Neslihan Kaya Terzi, İsmail Yılmaz, Ayşim Büge Öz

**Affiliations:** 1 Department of Pathology, İstanbul Sultan Abdulhamid Han Training and Research Hospital, İstanbul, Turkey; 2 Department of Pathology, İstanbul University Cerrahpaşa Faculty of Medicine, İstanbul, Turkey

**Keywords:** Genetic changes, grade II-III glial tumor, World Health Organization 2016 central nervous system classification

## Abstract

**Background:**

With the help of genetic studies, it is possible to obtain information about diagnosis and prognosis of glial tumors.

**Aims:**

To categorize the cases according to the new World Health Organization Central Nervous System classification by reconsidering the histologic features of oligodendrogliomas, astrocytomas and oligoastrocytomas. We also evaluated whether these genetic features have prognostic significance.

**Study Design:**

Diagnostic accuracy study.

**Methods:**

Between the years 2011 and 2016, 60 gliomas were examined. Archival material from the Department of Pathology was used for histopathological, immunohistochemical, and molecular analyses. All the cases were classified and graded according to the new 2016 World Health Organization criteria. IDH1 (R132H), alpha thalassemia/mental retardation syndrome, and p53 antibodies were applied immunohistochemically. The 1p/19q status and platelet-derived growth factor receptor-α/CEP4 amplification were evaluated by fluorescence in situ hybridization. After molecular tests, if the diagnosis of oligodendroglioma or astrocytoma is not diagnosed, case should be diagnosed as oligoastrocytoma. Sensitivity, specificity, positive predictive level, negative predictive level, and accuracy rate were evaluated in accordance with the specified threshold levels.

**Results:**

Except for 1 case (3.7%), all cases of grade 2 and grade 3 oligoastrocytoma were diagnosed with astrocytoma or oligodendroglioma without any change of grade. Except for 2 case (6.8%), all cases of grade 2 and grade 3 oligodendroglioma were diagnosed oligodendroglioma. All astrocytomas (100%) were given same diagnosis. There is no specific or sensitive test for the diagnosis of oligoastrocytoma. However, 1p/19q codeletion was spesific (100%) and sensitive (100%) for oligodendroglioma. ATRX and p53 mutation showed high spesificity (100% and 95.1% respectively) for diagnosing astrocytoma. Platelet-derived growth factor receptor-α/ CEP4 was not detected in any of the cases. There was association between isocitrate dehydrogenase mutation and 1p/19q loss with longer survival (respectively p=0.147 and p=0.178).

**Conclusion:**

In grade 2 and grade 3 glial tumors, pathological diagnosis is not possible only by histological examination. Overall, there was a diagnosis change in 28 cases (46.6%). Especially in cases of oligoastrocytoma, the diagnosis is changed by molecular tests.

Glial tumors are the most prevalent primary brain tumors. Diffuse glial tumors of the central nervous system are classified by the World Health Organization as astrocytomas, oligodendrogliomas, and mixed oligoastrocytomas according to histological findings (1). A new classification based on genetic evidence was published by World Health Organization in 2016. Molecular and cytogenetic examinations also produced results that further contributed to the diagnosis and prognosis of these tumors in addition to the classification performed mostly by histomorphological methods. Oligodendrogliomases have been found that the combined loss of 1p and 19q is closely associated with classical oligodendrogliomas morphology and better prognosis (2).

In glial tumors, point mutations at codon 132 in the isocitrate dehydrogenase (IDH) 1 gene (human cytosolic nicotinamide adenine dinucleotide phosphate hydrogen-dependent isocitrate dehydrogenase 1) may be observed. The most common mutation is R132H, and the IDH1 protein, which is the product of this mutation, can be visualized immunohistochemically. Another less common IDH mutation is IDH2, and it is another IDH isoform, which does not yet have commercial primer antibodies for immunohistochemistry, and can be demonstrated by DNA sequence analysis. Its most common mutation is R172W (3).

As constitute 10%-11% of glial tumors and it is known through the new classification that the genetic alterations that differentiate grade II-III astrocytic tumors from oligodendrogliomas are p53 and alpha thalassemia/mental retardation syndrome (ATRX) mutations (4). p53 mutation rate in all a grades is approximately 30%-50% (5). ATRX is located in chromosome Xq21.1 and encodes a protein that is part of the H3.3-ATRX-DAXX chromatin regulatory pathway (6). It is often found together with IDH mutation, and they exclude each other with loss of 1p/19q, which are very helpful in diagnosis. However, it is not yet clear whether it has an independent role from other molecular markers in terms of prognosis (7).

High grade astrocytomas are characterized by alterations in receptor tyrosine kinase signaling, and abnormal platelet-derived growth factor signaling has been demonstrated. Although there is no generally accepted predictive value of detecting platelet-derived growth factor receptor overactivity for glial tumors, this genetic alteration is of great diagnostic and prognostic importance (8).

Oligoastrocytomas tumors are still very controversial in the 2016 World Health Organization classification. They constitute 0.9% of all brain tumors and 3.3% of primary brain tumors. The diagnosis is oligoastrocytomas when oligodendroglial and astrocytic genetic alterations coexist (4).

In the 2016 classification, a group of tumors specified as “not otherwise specified (unspecified)” was included. “Not otherwise specified” is diagnosed when molecular parameters that are the genetic alterations in glioma cannot be examined or if no informative genetic alteration can be detected (4).

## MATERIALS AND METHODS

### Patients and samples

Between the years 2011 and 2016, 60 gliomas [19 oligodendrogliomas (31.6%), 10 anaplastic oligodendrogliomases (16.6%), 19 oligoastrocytomas (31.6%), 8 anaplastic oligoastrocytoma (13.3%), 2 astrocytoma (3%), and 2 anaplastic astrocytoma (3%)] were examined.

**Figure 1:**
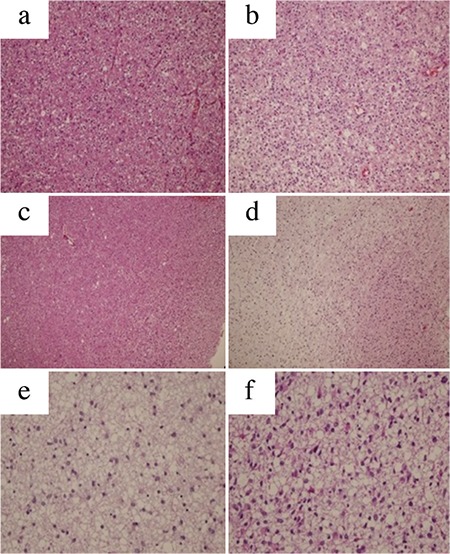
a-f. Microscopic photographs of specimens from histopathological diagnoses (Hematoxylin eosin, original magnifications: a, b: ×200; c, d: ×100; and e, f: ×400). Oligodendrogliom (a); anaplastic oligodendrogliomas (b); oligoastrocytoma (c); anaplastic oligoastrocytoma (d); astrocytoma (e); and anaplastic astrocytoma (f).

**Figure 2:**
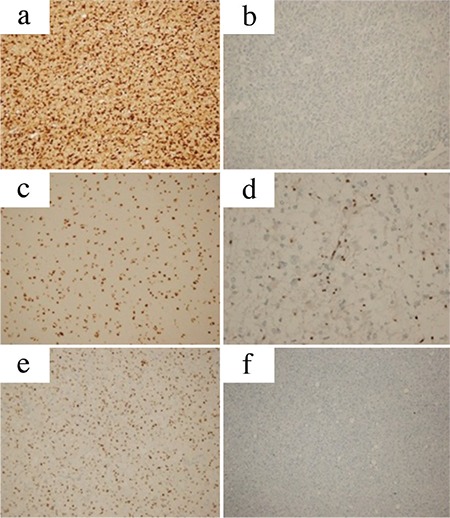
a-f. Microscopic photographs of immunohistochemical changes (original magnifications: a-e: ×200, d: ×400, and f: ×100). Cytoplasmic and nuclear positivity with isocitrate dehydrogenase1 (R132H) antibody (a). Negative with isocitrate dehydrogenase1 (R132H) antibody (b). Nuclear positivity (non-mutant) with alpha thalassemia/mental retardation syndrome antibody (c). Nuclear negativity (mutant) with alpha thalassemia/mental retardation syndrome antibody, positivity in vessel walls, and reactive glial cells (d). Nuclear positivity with p53 antibody (90%, score 3+) (e). Nuclear negativity with p53 antibody (0%, score 0) (f).

Archival material from the Department of Pathology was used for histopathological, immunohistochemical, and molecular analyses. Slides of all these cases were reviewed (Figures 1 and 2). All the cases were classified and graded according to the existing 2016 World Health Organization criteria.

The central ethics committee approved the study (2016/A18). The funding of the study was provided by the scientific research project unit of the hospital.

### Immunohistochemistry

Immunohistochemistry was carried out on at least one representative block in all the cases. Immunohistochemistry was performed using a primary antibody against the following antigens-IDH1 R132H (Dianova, dilution 1:40), ATRX (Sigma, dilution 1:300), and p53 (Dako, dilution 1:50). Cases showing cytoplasmic positivity for IDH1 in >10% of tumor cells were considered positive. Loss of nuclear staining for ATRX in tumor cells (>90%) was considered positive for ATRX mutation. Nuclear positivity for p53 in >50% of tumor cells was considered positive.

### Mutation analysis

**Figure 3:**
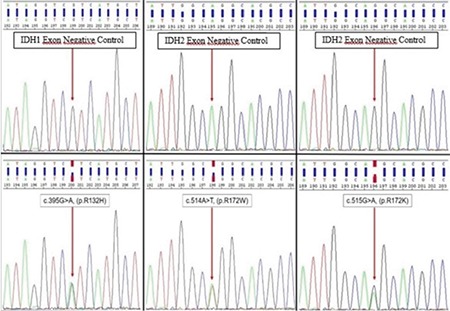
Examples of isocitrate dehydrogenase1 and isocitrate dehydrogenase2 mutations detected by the Sanger sequence analysis. In the upper image, isocitrate dehydrogenase1 and isocitrate dehydrogenase2 gene exogenous negative control for exon 4 and in the bottom image sample forward sequence electrophoresis of the mutated patients.

Mutations in exon 4 of IDH1 and IDH2 genes (well-known hotspot regions for oncogenic mutations) (Figure 3) were analyzed by polymerase chain reaction (PCR)-based direct sequencing using representative formalin-fixed paraffin-embedded tumor samples. DNA was extracted from the formalin-fixed paraffin-embedded tissue by a commercially available kit (50) (catalog #56404; QIAGEN, Hilden, Germany) as per manufacturer’s instruction. DNA concentrations of the samples were assessed spectrophotometrically using a Nanodrop 1000 spectrophotometer (Thermo Scientific, USA). PCR amplifications were performed in a Thermal Cycler (ABI, Applied Biosystems, USA) using HotStarTaq DNA Polymerase kit (catalog#203205; QIAGEN) and appropriate primers:

- IDH1 Forward: 5′CCAAGTCACCAAGGATGCTG′3

- IDH1 Reverse: 5′TCACATTACTGCCAACATGACTT′3

- IDH2 Forward: 5′CCGTCTGGCTGTGTTGTTG′3

- IDH2 Reverse: 5′AGTCTGTCGCCTTGTACTGC′3

PCR reactions were run as total volume of 50 μL reaction mixtures consisting of nuclease-free water, 5 μL 10× PCR Buffer, 2 μL 25 mM MgCl2, 1.5 μL 10 mM deoxyribonucleotide triphosphate mix (ABI, Applied Biosystems), 6 μL of each primer (4 pmol/μL), 0.25 μL of Hot Start Taq DNA polymerase, and 200 ng of each tumor DNA. After an initial denaturation at 95 °C for 15 min, 42 cycles were performed of 30 s denaturation at 95 °C, 30 s annealing at 58 °C, and 45 s extension at 72 °C, followed by a final extension of 10 min at 72 °C. The intensity of PCR products was checked by running 5 μL of each PCR reaction with 2 μL of loading dye on a 2% agarose gel. Reagent contamination control was achieved by examining lane for “No DNA” blank tube. Then, all succeeded PCR products were purified using the QIAquick PCR Purification Kit (catalog#28106; QIAGEN) according to the manufacturer’s instructions. The purified amplicons were submitted to direct sequencing in both directions (forward and reverse) using reagents from the Big Dye Terminator v3.1 Cycle Sequencing kit (ABI, Applied Biosystems) in accordance with the manufacturer’s protocol. After ethanol precipitation, subsequent products were run on the ABI-3730 (48 capillary) automatic sequencer (Applied Biosystems). Bidirectional sequence traces were analyzed with SeqScape® Software v3.0 (Applied Biosystems) and manually reviewed.

### Fluorescence in situ hybridization 1p/19q and platelet-derived growth factor receptor-α/CEP4

The fluorescence in situ hybridization (FISH) analysis was performed on 5-micron thick formalin-fixed paraffin-embedded tissue samples.

Deparaffinization, prehybridization, and hybridization steps have been done according to the datasheet. One hundred tumors cells were analyzed on a fluorescent microscope (Olympus BX61; Olympus Optical, Japan). The cells were captured on a computer system with a digital camera (XLMM, Dage-MTI, Indiana, USA) and compatible software (Duet®, Bioview Ltd., Israel).

**Figure 4:**
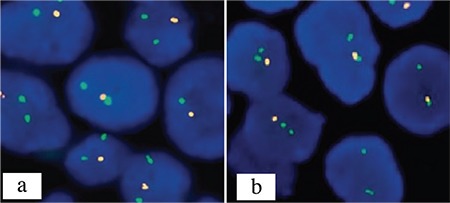
a, b. Microscopic photographs of 1p/19q fluorescence in situ hybridization studies. Oligodendrogliomas case showing 1p loss (red signal: 1p36 and green signal: 1q25) (a), Loss of 19q at the same time (red signal: 19q13 and green signal: 19p13) (b).

Dual-color paired probes for 1p and 1q (1p36 Spectrum Orange and 1q25 Spectrum Green, Vysis LSI probes, Abbott Molecular, Des Plaines, Illinois, USA) were hybridized simultaneously on one slide and similarly for 19q and 19p (19q13 Spectrum Orange and 19p13 Spectrum Green, Vysis LSI probes) on a separate slide. Using a fluorescence microscope with an oil immersion 100× objective, each hybridization was analyzed by locating an area with a high proportional density of neoplastic nuclei and evaluating scoring a minimum of 100 nonoverlapping interphase nuclei for the numbers of green and orange signals in each nucleus. With the probe pairs configured as they are, we calculated the proportion of orange (target chromosome arm) to green (comparison/control chromosome arm) signals. Based on laboratory experience, a proportion <0.80 represents a deletion. The ratio of SpectrumOrange to SpectrumGreen signals (total orange/total green) was calculated (Figure 4).

Platelet-derived growth factor receptor-α Break Apart was studied in chromosome 4q12 loci with separate bicolor probes (green and orange; Cytotest, RO, USA). The orange signal was obtained when 3′ of the target probes were hybridized in the 4q12 loci, whereas the green signal was obtained when 5′ of the target probes were hybridized. CEP4 4p11-q11 aqua probe was added as the control probe. Aqua signal was obtained by centromeric region hybridization. In normal (negative) cells, two aqua and two fusion yellow (orange + green) signals were observed.

**Figure 5:**
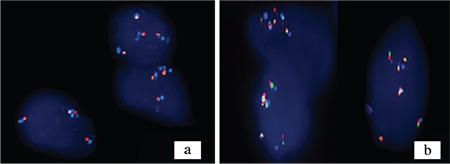
a, b. Fluorescence in situ hybridization platelet-derived growth factor-α/CEP4 study; photographs of anaplastic astrocytoma cases that detected (a), low-level polysomy and high-level polysomy (b).

In an abnormal cell with an increase in the number of copies in the 4q12 loci, more than two fusion yellow and aqua signals were observed. If all three signals increase at the same time and the number of cells with >2 and <6 signals is more than 10%, polysomia is detected. Polysomia-detected cases were separated into two groups: three to four signals were evaluated as low-level polysomia and five to six as high-level polysomia (Figure 5). Platelet-derived growth factor receptor-α/CEP4 centromere ratio was assessed.

Amplification: If the platelet-derived growth factor receptor-α/CEP4 signal ratio was ≥2, it was evaluated as amplification (9).

### Statistical analysis

In our study, Statistical Package for the Social Sciences version 21.0 (Chicago, Illinois, USA) was used for statistical analysis of data. Survival using the Kaplan-Meier method was calculated based on the date of initial surgery and pathological confirmation and the date of death. The results were evaluated in 95% confidence interval.

Sensitivity, specificity, positive predictive level, negative predictive level, and accuracy rate were evaluated in accordance with the specified threshold levels. The significance value was regarded as p<0.05.

We performed a post-hoc power analysis by G*Power 3.1.9.2. While type II error (β) probability was observed less than 0.01 (power >0.99) for the combined diagnostic algorithm and single ATRX, p53 mutations and 1p/19q deletions but less than 0.40 (power <0.60) for IDH mutation and CEP4 amplification.

## RESULTS

The demographic and clinical characteristics of the 60 patients are shown in Table 1.

**Figure 6:**
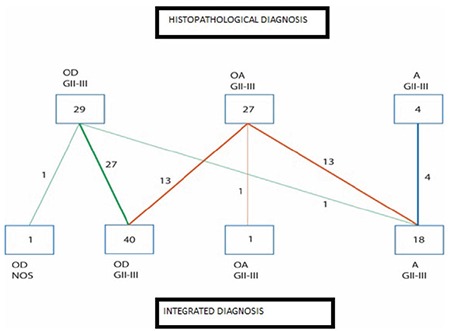
Number of cases showing the transformation of histopathological diagnosis (first diagnosis) into integrated diagnosis (new diagnosis) after molecular testing. A: astrocytoma; OA: oligastrocytoma; OD: oligodendroglioma; GII-III: grade II-III

Histopathologically, 19 cases were diagnosed with oligodendrogliomas (31.6%), 10 with anaplastic oligodendrogliomas (16.6%), 19 with oligoastrocytomas (31.6%), 8 with anaplastic oligoastrocytomas (13.3%), 2 with A (3%), and 2 with anaplastic astrocytoma (3%). We reclassified all of the cases according to the genetic alterations detected. Overall, there was a diagnosis change in 28 cases (46.6%) (Figure 6). One case was left as anaplastic oligoastrocytomas since it had both 1p/19q loss and ATRX mutation.

Immunohistochemical analyses using IDH1 (R132H) antibody showed positive results in 52 cases (86.6%) and negative results in 8 cases (13.3%).

A guanine-to-adenine point mutation in the IDH1 (R132H) region was detected by sequencing in 37 out of 52 cases immunohistochemically expressing IDH1, but no mutation was detected in 4 cases by sequencing although IDH1 expression was immunohistochemically confirmed in repeated experiments. In eight cases in which IDH1 expression was not detected via immunohistochemical analysis, the point mutation in IDH1 (R132H) region was detected by sequencing in three cases, and guanine to adenine and thymine to adenine change in IDH2 R172K and R172W regions were detected by sequencing in two cases. In the remaining three cases, no IDH mutation was found by immunohistochemical methods or Sanger sequence analysis.

ATRX mutation and 1p/19q loss are mutually exclusive genetic alterations (p<0.001; Table 2).

In the evaluation of CEP4 polysomia, polysomia was detected in 13 out of 50 cases with a positive signal (26%). Seven (53.8%) of these cases had high-level polysomia, whereas six (46.1%) had low-level polysomia. CEP4 amplification showed sensitivity, specificity, positive predictive level, and negative predictive level at 42.8%, 79.4%, 46.1%, and 77.1%, respectively, for astrocytoma (Table 3).

For predicting glial tumor, the sensitivity, specificity, positive predictive level, negative predictive level, and the accuracy of the cutoff value for ATRX mutation, p53 mutation, and 1p/19q codeletion are indicated in Table 3. ATRX mutation showed a sensitivity of 72.2%, a specificity of 100%, a positive predictive level of 100%, and an negative predictive level of 89.1% for astrocytoma (p=0.002). p53 mutation showed a sensitivity of 77.7%, a specificity of 95.1%, a positive predictive level of 87.5%, and an negative predictive level of 90.6% (p=0.005). For predicting oligodendroglial tumor, the sensitivity, specificity, positive predictive level, and negative predictive level of the cut-off value for 1p/19q codeletion was 100% (p<0.001).

**Figure 7:**
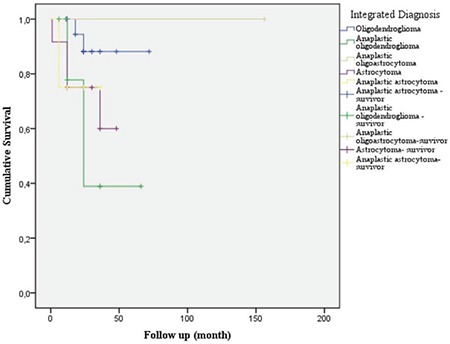
Survival times according to integrated diagnosis. Oligodendrogliomas had the best survival rates (%33.3 cases survived in 5 years) and the cases with the worst survival were diagnosed with anaplastic oligodendrogliomas (%16.7 cases survived in 5 years), astrocytoma (all of them died in 5 years) and anaplastic astrocytoma (all of them died in 5 years).

The cases with the best survival rates were diagnosed with oligodendrogliomas, and the cases with the worst survival were diagnosed with A, anaplastic astrocytoma, and anaplastic oligodendrogliomas (Figure 7). There was no statistically significant difference (p=0.901).

**Figure 8:**
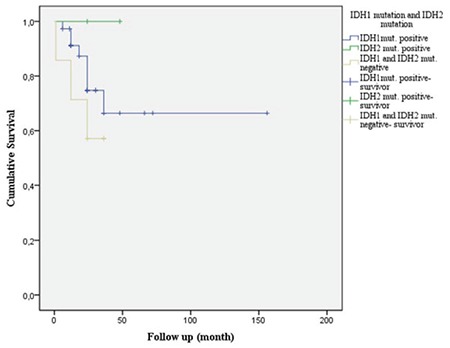
Survival in cases which isocitrate dehydrogenase 1 and isocitrate dehydrogenase 2 mutations are detected and mutations are not detected. %16.7 cases of IDH1 mutation positive cases survived in 5 years. Any case of without IDH1 mutation didn’t survive in 5 years. One case of IDH2 mutation (%12.5) survived in 5 years.

**Figure 9:**
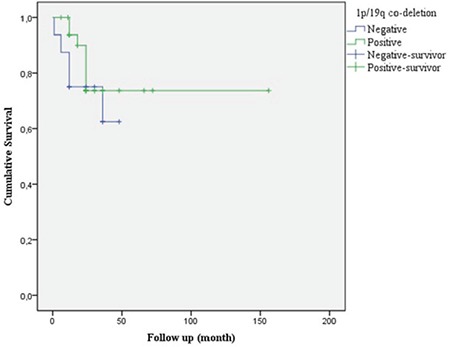
Survival with and without 1p/19q loss. Survival rate was 64.3% in the group without 1p / 19q and 81.6% in the group with 1p / 19q codeletion. This difference was not statistically significant.

The survival time of cases with IDH mutation with 1p/19q loss was longer (p=0.174 and p=0.178, respectively; Figures 8 and 9).

A survival rate of 76.2% was observed in cases with negative PGFR-α amplification. A survival rate of 74.2% was observed in cases with negative CEP4 polysomia and 75% in cases with positive CEP4 polysomia (p=0.966).

## DISCUSSION

In our study, molecular examinations were performed in accordance with the criteria of the 2016 World Health Organization central nervous system tumor classification recommended for grade II and grade III oligodendrogliomases, As, and oligoastrocytomaes, and new integrative diagnoses were established. Diagnosis changed completely in 28 out of 60 cases evaluated in this study (46.6%).

The aim of this study was to investigate the contributions of 1p/19q loss typically observed in oligodendroglial tumors, and ATRX mutation and p53 expression observed in astrocytic tumors and IDH mutation, which is known to have prognostic value in glial tumors and to rediagnose cases according to the new classification. In addition, platelet-derived growth factor receptor-α/CEP4 amplification, which is more frequently seen in high grade astrocytoma, was also included in the study to assess its implications in pathology applications.

In older classifications, oligoastrocytomas diagnosis was very common, whereas in the new classification, oligoastrocytomas diagnosis covers a very small area. There are still cases diagnosed with oligoastrocytomas (1).

Similar to our study, Sahm et al. (10), which looked into 43 oligoastrocytomas cases, assessed 1p/19q, IDH, p53, and ATRX status of cases. Based on the results of the study, loss of 1p/19q, ATRX mutation, and p53 nuclear expression were observed simultaneously in one case. This case was described as a molecular hybrid since it exhibited genetic alterations specific to oligodendrogliomases and strocytomas (10). In another study, ATRX expression and loss of 1p/19q were simultaneously observed in only 3 of 1.041 glial tumors (11). IDH1 mutation is the first genetic event during gliomagenesis in oligodendrogliomases and strocytomas. Following the IDH mutation, different genetic alterations occur depending on tumor-specific differentiation and behavior pattern (12). IDH mutation has been an important area of diagnosis and prognosis and contributes greatly to routine pathological studies. The capability to examine mutation assessments by immunohistochemistry has enabled these assessments to be rapidly included in routine pathology practices. Parsons et al. (13) investigated IDH mutation in 22 cases of glioblastoma and called attention to this topic. A more recent study of 1.010 glial tumors from six centers examined the role of IDH mutation in glial tumorigenesis in general (14).

Today, it is known that 90% of mutations in IDH gene are IDH1 (R132H) mutations (13). IDH1 mutation rates are reported as 42.9%-100% in oligodendrogliomas, 46%-100% in anaplastic oligodendrogliomas, 74%-90% in A, and 27.3%-73% in anaplastic astrocytoma (15).

In our study, IDH1 (R132H) expression was detected in 23 (92%) oligodendrogliomas cases, 12 (80%) anaplastic oligodendrogliomas cases, 1 (100%) oligodendrogliomas NOS case, 12 (85.7%) A cases, 3 (75%) anaplastic astrocytoma cases, and 1 (100%) anaplastic oligoastrocytomas case. The incidence of IDH1 (R132H) expression that we detected in all intermediate-grade glial tumors included in the study is consistent with the high values reported in the literature (15).

There are commercially available primary antibodies for IDH1 (R132H) mutation assessment, and no commercial antibodies are available for other commonly identified IDH1 regions such as IDH1 R132G, R132S, and R132C and for IDH2 assessment.

Consistent with the literature, there are cases in our study in which immunohistochemical IDH1 expression was not detected, whereas mutation in IDH1 (R132H) was detected in the same region following Sanger sequence analysis (12). These results suggest that the specificity of the antibody used may not be high. Therefore, if there is a strong suspicion of false-negative IDH1 cases based on immunohistochemical assays, IDH mutation should be properly assessed using other methods.

IDH2 mutation is less frequent than IDH1 mutation, and they are mutually exclusive (3). In our study, IDH1 and IDH2 mutations were not observed simultaneously when DNA sequence analysis was performed.

Assessing 1p/19q loss in oligodendroglial neoplasias is an important aid in establishing a diagnosis (2). Cairncross et al. (16) were the first ones to report that the combined loss of chromosome 1p/19q was predictive for chemotherapy response and an effective prognostic factor for longer survival in anaplastic oligodendrogliomas patients.

The loss rates of 1p and 19q that we observed in oligodendroglial tumors are consistent with those reported in the literature (17). Our results confirm the hypothesis that there are no oligodendroglial tumors without 1p/19q loss (4).

In our study, we found a statistically negative correlation between ATRX mutation and 1p/19q loss (p<0.001).

Platelet-derived growth factor receptor-α/CEP4 amplification and increase in gene copy numbers were assessed by FISH method in all cases, and no amplification was detected. CEP4 polysomia was detected mostly in astrocytic high-grade tumors, consistent with the literature (18). In high grade astrocytomas, platelet-derived growth factor receptor-α amplification is common and can manifest as high-level or low-level amplifications. Platelet-derived growth factor receptor-α amplification increases with grade and is associated with a less favorable prognosis (19). Although biological, this is probably more accurately described as a “high-level polysomy” rather than true gene amplification; the definition, nonetheless, correlates strongly with clinical outcome, including therapeutic response to tyrosine kinase inhibitors (20).

When survival rate was assessed according to the diagnoses of the cases, the best survival rate was obtained in oligodendrogliomas cases.

Several studies have demonstrated that survival is better in oligodendrogliomases than in low-grade As. In a study conducted in 987 cases, median survival time was 11.6 years in oligodendrogliomas and 5.6 years in low-grade As. These values are similar for grade III tumors; a study conducted by the European Cancer Treatment and Research Organization-Brain Tumor Group, which included 368 patients, demonstrated that median survival time was 34.7 months in anaplastic oligodendrogliomas (21).

IDH mutant tumors have better prognosis regardless of grade (12,22). In our study, the survival rate was higher in cases with IDH mutation, but no statistical difference was there (p=0.147).

The presence of 1p/19q loss has been associated with long overall survival in patients regardless of treatment (21). In our study, the survival rate was 64.3% in cases without 1p/19q loss and 81.6% in cases with 1p/19q loss (p=0.178).

In summary, molecular glioma research has significantly advanced the understanding of glioma pathogenesis and identified a number of diagnostic, prognostic, and/or predictive molecular markers that currently are on their way into clinical application. The presence or absence of IDH mutations, ATRX mutation, p53 mutation, and 1p/19q codeletion can be used to define gliomas with characteristic distributions of clinical behavior, acquired genetic alterations, and associated germline variants. This framework could be further refined through the incorporation of alterations in platelet-derived growth factor receptor alpha or other alterations that might be useful to consider in the diagnosis of glioma.

## Figures and Tables

**Table 1:**
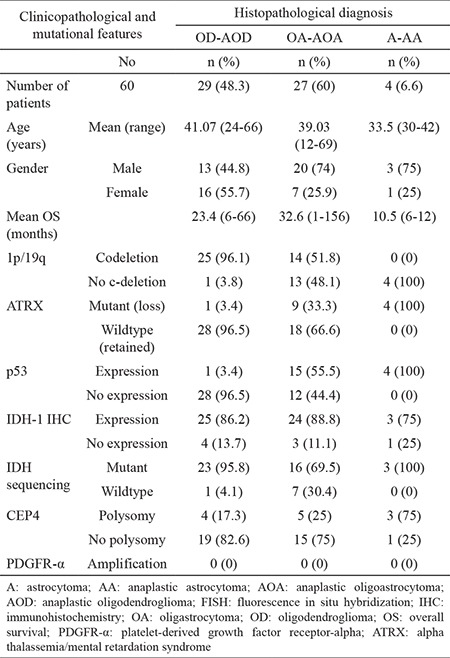
Clinical and molecular characteristics of the cases

**Table 2:**
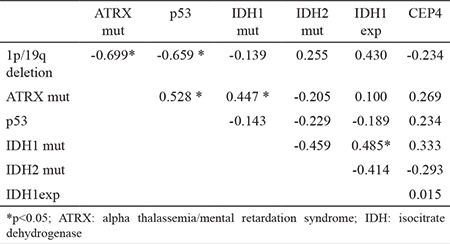
Correlation of the genetic changes in the study

**Table 3:**
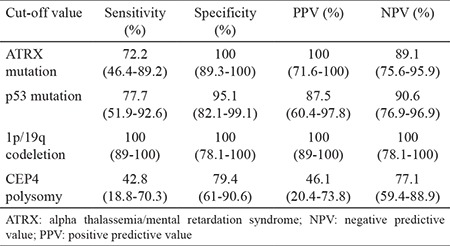
The sensitivity, specificity, positive predictive value, negative predictive value, and the accuracy of the cutoff values of ATRX mutation, p53 mutation, and CEP4 polysomy for astrocytoma and 1p/19q codeletion for oligodendroglioma
